# Multiple transitions in sick leave, disability benefits, and return to work. - A 4-year follow-up of patients participating in a work-related rehabilitation program

**DOI:** 10.1186/1471-2458-12-748

**Published:** 2012-09-06

**Authors:** Irene Øyeflaten, Stein Atle Lie, Camilla M Ihlebæk, Hege R Eriksen

**Affiliations:** 1 The National Centre for Occupational Rehabilitation, Haddlandvegen 20, 3864, Rauland, Norway; 2 Uni Health, Uni Research, Bergen, Norway; 3 Health UMB, IHA, University of Life Sciences, Ås, Norway; 4 Department of Health Promotion and Development, University of Bergen, Bergen, Norway

**Keywords:** Sick leave, Return to work, Employment, Register data, Follow-up, Occupational, Vocational rehabilitation

## Abstract

**Background:**

Return to work (RTW) after long-term sick leave can be a long-lasting process where the individual may shift between work and receiving different social security benefits, as well as between part-time and full-time work. This is a challenge in the assessment of RTW outcomes after rehabilitation interventions. The aim of this study was to analyse the probability for RTW, and the probabilities of transitions between different benefits during a 4-year follow-up, after participating in a work-related rehabilitation program.

**Methods:**

The sample consisted of 584 patients (66% females), mean age 44 years (sd = 9.3). Mean duration on various types of sick leave benefits at entry to the rehabilitation program was 9.3 months (sd = 3.4)]. The patients had mental (47%), musculoskeletal (46%), or other diagnoses (7%). Official national register data over a 4-year follow-up period was analysed. Extended statistical tools for multistate models were used to calculate transition probabilities between the following eight states; working, partial sick leave, full-time sick leave, medical rehabilitation, vocational rehabilitation, and disability pension; (partial, permanent and time-limited).

**Results:**

During the follow-up there was an increased probability for working, a decreased probability for being on sick leave, and an increased probability for being on disability pension. The probability of RTW was not related to the work and benefit status at departure from the rehabilitation clinic. The patients had an average of 3.7 (range 0–18) transitions between work and the different benefits.

**Conclusions:**

The process of RTW or of receiving disability pension was complex, and may take several years, with multiple transitions between work and different benefits. Access to reliable register data and the use of a multistate RTW model, makes it possible to describe the developmental nature and the different levels of the recovery and disability process.

## Background

Return to work (RTW) and sick leave are often used as a measure of health and recovery after work-related rehabilitation programs [1,2]. However, the way RTW is measured varies between studies, and comprises different measures like; proportion of individuals that are working, number of sick-leave days or number of sick leave episodes, at follow-up. Thus there is no clear agreement among researchers about how sick leave and successful RTW should be measured [1,3]. Furthermore, when analysing and presenting data on RTW, there are several conceptual and methodological challenges [1,2,4]. For example, working part-time may be a common outcome after sick leave and not always asked for or sufficiently considered in the analyses and presentations. Practice and legislation on partial social security benefits differ between countries and make comparisons difficult, and may necessitate improved or more harmonized sick leave data, at least in the Nordic countries [5]. RTW is a complex, and sometimes long-lasting process, where the individuals may be in multiple and recurrent states; i.e. receiving different social security benefits or working, and over time they may shift between these states [2]. Therefore analyses of multiple longitudinal and repeated events are required for better understanding of the RTW-process [3,6].

The costs and burdens of long-term sick leave are substantial both for the individual, the employer and society [3,7]. Medical diagnoses related to mild or moderate mental health problems and musculoskeletal conditions, are the most common diagnoses for long term sick leave and disability pension in Norway [8-11], and in other European countries [12]. These diagnoses are often characterized more by symptoms and distress, than by consistent demonstrable tissue abnormalities [13,14], and are often based on the patients’ own reports of pain and complaints [15,16], and may be classified as subjective health complaints [13,15,16]. For some it may be experienced as an increased burden when there are no objective findings to explain their symptoms [17]. Research on work-related rehabilitation emphasize the importance to be seen, heard and taken seriously by the professionals, for individuals on long-term sick leave [18]. The increase in long-term sick leave and disability pension is not due to ill health alone, but could be related to changes in working life and health expectations [8]. Length of the sick leave and the possible burden of being out of work should also be emphasized in the outcome measures, made visible in form of transitions and shifts [1,12,19]. Further when the purpose of a study is to analyse the effects of different treatments or rehabilitation efforts, partial recoveries should also be considered, along with full-time return to work [1,19]. There is still limited knowledge of the long-term occupational status for patients after participation in work-related rehabilitation [3,20]. This may in part, be due to the different challenges described above. Outcome measures in follow-up studies on RTW tend to distinguish only between those working full-time and those not working at all, at a particular point in time [19].

In a follow-up study of low back pain patients, Lie and colleagues [1] applied statistical tools for multi-state models, synthesizing the transition intensities between three different states the patient could be in, after an intervention; recovery (return to work), sick leave benefits, or disability pension. In this article we have extended the model to include eight different categories for sick leave benefits or return to work. Data on different partial or full-time benefits were obtained from national registers of The Norwegian Labour and Welfare Administration. In this study we included patients on long-term sick leave benefits that were diagnosed primarily with mental and musculoskeletal diagnoses. The objective of the study was to describe the complexity of the RTW course in a 4-year follow-up period after an inpatient, interdisciplinary work-related rehabilitation program, analysed with tools for multi-state models [2,21,22].

## Methods

### Participants

584 patients (383 women (66%) and 201 men (34%), mean age 44 years, (SD = 9.3); age range 22 – 66 years) that had participated in a 4-week inpatient work-related rehabilitation program in 2001, were included in the study. All the participants were on different sick leave benefits (mean duration 9.3 months (SD = 3.4) range: 0 to 61 months) before their arrival at the rehabilitation clinic, and had a mental (47%), or a musculoskeletal (46%) diagnosis. 7% had other diagnoses. 25% had received benefits for more than 12 months. During the last year before arrival to the rehabilitation clinic, the majority had only one sick leave episode (79%), and the maximum number of sick leave episodes was three. Most of the participants were on full-time sick leave (71%), but 17% were on partial sick leave before inclusion.

The patients ended their stay at the rehabilitation clinic between January 14, and December 23, 2001. The continuous register data was obtained for all participants from departure until December 30, 2004. During the 4-year follow-up period 6 participants died, 2 received early retirement pension, and 2 participants had passed the age of 67 years and received ordinary retirement pension, at which time these observations were censored.

All of the participants had given informed consent to participate in a follow-up study. All principles in the Helsinki declaration were followed. The Medical Ethics Committee, Region South, in Norway, and the National Social Science Data Service approved the project.

### Interdisciplinary work-related rehabilitation

The intervention program was a 4 week, inpatient, interdisciplinary rehabilitation program, taking place at a national occupational rehabilitation clinic. A combination of individual and group based interventions, which included physical activity, education, and cognitive behavioural modification were offered. The goal of the program was to improve the level of physical and mental functioning, improve the ability to work, and increase the likelihood of return to work for individuals on long-term sick leave benefits. Before finishing the rehabilitation program, a detailed RTW plan was developed in a collaborative effort between the patients and the interdisciplinary team. This plan could include participation from several stakeholders outside the rehabilitation setting, e.g. different health care providers, the work place, and the local Labour and Welfare Administration office, including the labour marked agency.

### Measurements

Socio-demographic data was obtained from the patient journals, and included gender, age, medical diagnoses and baseline-data regarding sick leave length and different applied benefits in a certain period, before the patients were enrolled into the rehabilitation program. In this study we only had access to the main diagnoses; however it is recognized that the majority of patients had several diagnoses. The socio-demographic data was combined with register data from The Norwegian Labour and Welfare Administration. The disbursements to individuals on different benefits are based on these registers, therefore the obtained data is considered to be complete. The register included data on each individual, with all benefits, which included a start, and end date on each benefit. Since the register contains sparse data on whether a person is actually working or not, work was defined as the time gaps with no benefits. Unfortunately we have no information if these time gaps could be unemployment benefits.

In Norway the employer pays the compensation for the first 16 days of a sick leave period, thereafter The Labour and Welfare Administration pays it. Sick leave days compensated by employers were not included in these analyses. During the first year on sick leave, the compensation constitutes 100% of the work salary. If the employee has not RTW after one year, the individual on sick leave will receive a rehabilitation allowance, either medical or vocational rehabilitation, which constitutes approximately 66% of the salary. To be eligible for medical rehabilitation allowance, there must be a certain probability to recover after medical treatment. Vocational rehabilitation allowance is granted for individuals that may benefit from vocational guidance to RTW, e.g. work training or professional re-education. An employee cannot be discharged due to sick leave; these legislations are especially strict during the first 12 months. In the Norwegian welfare system it is possible to be working part-time and at the same time receive social security benefit. Partial sick leave and partial disability pensions are the most common combinations with part-time work. Disability pension with a time limit from one to four years was introduced in Norway in January 2004, and was phased out in 2010.

### Main outcome

The main outcome in the study was being in any of the following eight states or benefits; 1) working, 2) partial sick leave or partial medical rehabilitation allowance, 3) full-time sick leave, 4) medical rehabilitation allowance, 5) vocational rehabilitation allowance, 6) partial disability pension (both time-limited and permanent), 7) time-limited disability pension, and 8) full-time permanent disability pension (see Table 1). The part-time sick leave included partial sick leave benefits from 20 to 90 percent, whereas for part-time rehabilitation allowance and disability pension it is a 50% lower limit.

**Table 1:** Abbreviations and definitions of the different benefits and working used in tables and figures through the manuscript

	
**8) DP**	Full-time permanent disability pension
**7)TLDP**	Time-limited disability pension
**6)PDP**	Partial disability pension (both time-limited and permanent)
**5)VR**	Vocational rehabilitation allowance
**4)MR**	Medical rehabilitation allowance
**3)SL**	Full-time sick leave
**2)PSL**	Partial sick leave and partial medical rehabilitation allowance
**1)W**	Working (no registered benefits)

### Statistical methods

The official register data included separate data files on sick leave, medical rehabilitation allowance, vocational rehabilitation allowance, time-limited disability pension and permanent disability pension. The files were merged together to form one complete event history (counting process) file, however overlap could occur between start and end date for some registered benefits in this merged file. We considered the benefits and working as ranked in accordance with Table 1. Thus individuals registered simultaneously on several overlapping benefits were considered to be in the highest ranked state according to Table 1; e.g. an individual registered with DP, and that at the same time was registered with a sick leave benefit, was considered to be on DP. Ideally overlap in the registers should not occur for full-time benefits; however combinations of partial benefits may occur in the registers when appropriate. Due to the ranking, we have not included data from the state with the lowest rank in the analysis; e.g. an individual registered on PDP, which at the same time was also registered on 100% medical rehabilitation allowance, was considered to be on PDP. This means also that for all partial benefits, we have not included information from the other registered benefit, thus individuals could be at work or on other partial benefits with a lower ranking according to Table 1.

Extended statistical tools for multi-state models were used to calculate conditional transition probabilities between the different states. The calculation of the conditional transition probabilities was based on non-homogenous Markov chains, calculated from non-parametric transition intensities, according to Aalen and Johansen [23]. The conditional transition probabilities give the probability to be in either of the eight states at follow-up, conditioned on which state the individual was in at baseline. The unconditional state probabilities gives the probabilities to be in either of the eight states at follow-up, regardless of which state the individual was in at baseline, also taking into account that all possible pathways and transitions between the different states are possible during the follow-up. The data was analyzed using custom made routines in the statistical package “R” [23,24].

## Results

Only 10% of the participants returned directly to full-time work after they had completed the rehabilitation program, i.e. no registered benefit in the official registers. The proportion of participants that returned to work, increased rapidly to 40% during the following year (see Table 2). The increase continued up to 51% at 4-year follow-up. A total of 71% of the participants were on sick leave immediately after departure from the rehabilitation clinic; 20% of these were on partial sick leave. There was a substantial decrease in participants on full-time sick leave, from 52% at departure to 4% at one year follow-up. After two years follow-up, the annual proportion on sick leave had increased to 8%. At 4-years follow-up 3% of the participants were on full-time sick leave. For annual proportions on the other benefits, see Table 2.

**Table 2:** Annual proportions (%) and numbers (n) of individuals working and on different benefits after departure from the rehabilitation clinic

	**Working#**	Partial* sick leave	**100% Sick leave**	**Medical rehab.**	**Vocational rehab.**	Partial** disability	Time-limited*** disability	**100% Permanent disability**	**Total**
	**% (n)**	**% (n)**	**% (n)**	**% (n)**	**% (n)**	**% (n)**	**% (n)**	**% (n)**	**n**
**Departure**	10.1 (59)	19.5 (114)	51.5(301)	11.0 (64)	5.3 (31)	2.1 (12)	0 (0)	0.5 (3)	584
**1 year**	39.7 (232)	10.6 (64)	3.5 (22)	19.2 (115)	21.1 (107)	3.7 (21)	0 (0)	3.2 (18)	579
**2 years**	40.6 (241)	7.8 (45)	7.6 (46)	11.5 (68)	19.5 (101)	6.4 (37)	0 (0)	6.6 (38)	576
**3 years**	45.1 (263)	5.7 (34)	4.1 (23)	5.7 (34)	18.1 (96)	9.7 (56)	1.9 (10)	9.7 (56)	572
**4 years**	50.7 (291)	2.7 (17)	2.8 (17)	1.9 (12)	14.8 (80)	10.8 (62)	3.8 (22)	12.5 (72)	573

# No registered benefits, * Partial sick leave and partial medical rehabilitation allowance, ** Partial time limited disability pension and partial permanent disability pension, *** Time-limited disability pension was introduced in Norway in January 2004.

**Figure 1:**
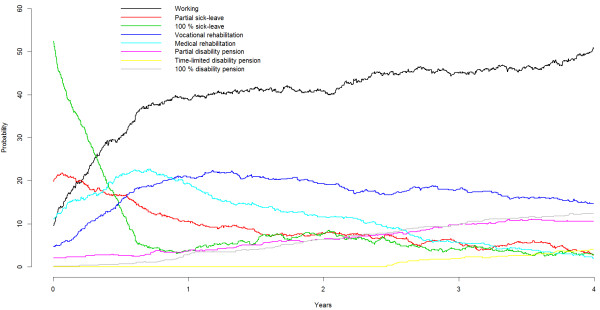
Probabilities for being on different benefits and working (state probabilities) during the 4-year follow-up.

During the 4-year follow-up the probability for working increased and the probability for being on sick leave decreased (see Figure 1 and Table 2). There was also an increased probability to be on disability pension, both partial, time-limited, and permanent.

When summing up cumulative days within each of the different benefits and working during the 4-year follow-up, the participants worked an average of 2.2 years (95% CI, 2.08-2.32). The average time on partial sick leave was 4.8 months (95% CI, 4.20-5.40), and 3.6 months (95% CI, 3.24-3.96) on full-time sick leave. The average time on medical rehabilitation allowance was 6 months (95% CI, 5.28-6.72), and 9.6 months (95% CI, 8.40-10.80) for vocational rehabilitation allowance. For disability pensions, the average time on partial disability was 4.8 months (95% CI, 3.84-5.76) and 6 months on full-time disability pension (95% CI, 4.80-6.72).

The probability of working at different points in time during the follow-up was not related to the benefit the participant was receiving when they departed from the rehabilitation clinic (see Table 3). For those working (i.e. no registered benefit) at departure, the probability for working after 1 year was 52%, and it was 54% at 4-year follow-up. Those that were on partial sick leave at departure were more likely to work during follow-up, compared to those that were on full-time sick leave, but this was only during the first years, and the confidence intervals were wide. Those receiving partial disability pensions at departure were more likely to work, compared to those on full-time disability pension. However the numbers of patients on these benefits at departure was very low, thus these results should be interpreted with caution. For those receiving medical rehabilitation allowance or vocational rehabilitation allowance at departure, it was an increased probability for working over time.

**Table 3:** The calculated multi-state probabilities for working in the 4-year follow-up, given the participants registered benefit or working at departure from the rehabilitation clinic

**Benefit at departure**	**at 1 year**	**at 2 years**	**at 3 years**	**at 4 years**
	**% (CI)**	**% (CI)**	**% (CI)**	**% (CI)**
Working#	52.4 (46.0-59.1)	46.7 (40.4-53.1)	49.1 (43.0-55.1)	53.8 (48.2-60.1)
Partial sick-leave*	49.1 (37.1-61.4)	50.2 (35.5-65.0)	52.4 (35.6-69.2)	56.2 (33.1-80.9)
Full-time sick-leave	38.4 (18.0-59.1)	40.3 (26.1-54.5)	44.7 (24.4-65.0)	50.6 (27.1-74.5)
Medical rehabilitation	28.4 (20.2-37.1)	33.5 (22.3-45.1)	39.0 (22.6-55.4)	45.8 (18.1-74.2)
Vocational rehabilitation	15.6 (8.7-22.5)	25.1 (16.6-33.5)	36.0 (26.4-46.0)	45.9 (35.1-57.0)
Partial disability**	6.7 (0–17.3)	15.3 (3.7-27.1)	20.8 (10.1-31.4)	27.7 (16.6-39.1)
Time-limited disability***	-	-	-	0.1 (0.0-1.5)
Full-time permanent disability	0.1 (0.0-1.3)	6.3 (0.0-14.2)	6.3 (0.0-13.1)	8.3 (1.9-15.0)

Percent and 95% Confidence Interval (CI) reported.

# No registered benefits, * Partial sick-leave and partial medical rehabilitation allowance, ** Partial time limited disability pension and partial permanent disability pension, *** Time-limited disability pension was introduced in Norway in January 2004.

There was an average of 3.7 transitions between different benefits and working, during the 4-year follow-up. The median number of transitions was 3.0. The minimum number of transitions for a single patient was zero and the maximum number was 18 transitions. 10 individuals had no registered transitions after leaving the rehabilitation clinic, thus they kept the same benefit as they had during their stay at the rehabilitation clinic. 4 of these were registered with vocational rehabilitation allowance and two were receiving disability pension. The last four were not registered on any benefit; thus they were defined as if they were working from the day they left the rehabilitation clinic.

For full-time sick leave 61% of the transitions were to work and 31% of the transitions were to medical or vocational rehabilitation allowances (see numbers in Table 4). From partial sick leave a total of 86% of the transitions were to work. The main pathway to permanent disability pension was through rehabilitation allowances; a total of 54% transitions, while for partial disability pension the main pathway was from work; 45% of the transitions, and from partial sick leave; 35% of the transitions.

**Table 4:** Number of individuals shifting between different benefits and work* during the 4-year follow-up period, n = 584

		**To W**	**To PSL**	**To SL**	**To MR**	**To VR**	**To PDP**	**To TLDP**	**To DP**	**Sum from**
**From**	**W**	0	188	309	42	66	38	3	22	**668**
**From**	**PSL**	308	0	2	2	13	30	1	3	**359**
**From**	**SL**	391	43	0	141	60	3	0	4	**642**
**From**	**MR**	127	0	1	0	98	7	17	34	**282**
**From**	**VR**	117	4	2	31	0	5	5	10	**174**
**From**	**PDP**	3	5	11	1	4	0	0	6	**30**
**From**	**TLDP**	0	0	0	0	0	0	0	2	**2**
**From**	**DP**	4	0	0	0	1	2	1	0	**8**
	**Sum to**	**950**	**240**	**325**	**217**	**240**	**85**	**27**	**81**	**Total transitions: 2165**

*W = working (no registered benefits), PSL = partial sick leave and partial medical rehabilitation allowance, SL = full-time sick leave, MR = medical rehabilitation allowance, VR = vocational rehabilitation allowance, PDP = partial time-limited disability pension and partial permanent disability pension, TLDP = time limited disability pension (introduced in Norway in January 2004), DP = full-time permanent disability pension.

During the follow-up period there was a total of 2165 registered transitions (see Table 4). The most frequent transitions were to and from work (950 and 668 transitions respectively). The second most frequent transition was from and to full-time sick leave. The single most frequent transition to work during follow-up was from being on full-time sick leave. The most frequent transition to medical rehabilitation allowance was also from full-time sick leave (141 transitions) while the most frequent transition to vocational rehabilitation was from medical rehabilitation (98 transitions). A total of 325 transitions were into partial benefits, of these 240 were partial sick leave/medical rehabilitation allowance, and 85 were partial disability pension, both time-limited and permanent. During the follow-up, a total of 81 transitions were to permanent disability pension. It was a total of 63 transitions from work to disability pension, either partial, time limited or permanent. Additionally for disability pension, time gaps in the registers were recorded; 3 transitions from partial and 4 transitions from permanent disability pension, indicating return to work (see Table 4).

## Discussion

Using statistical tools for multistate models in the analysis of official register data on different sick leave benefits made it possible to describe the composite process of RTW in detail. During a 4-year follow-up after a group of patients had finished a rehabilitation program, the average number of transitions between different benefits and work was 3.7 times. The maximum number of transitions for one individual was 18. This shows that the RTW process for people on long-term sick leave benefits, with non-specific musculoskeletal and mental health problems, may be long and complex. In addition to the high number of transitions between different benefits, the proportion of individuals that returned to full-time work, i.e. no registered benefit, increased from about 10% at departure from the rehabilitation clinic to above 50% throughout the follow-up period. It seem to be a trend that those being on partial sick leave at departure from the rehabilitation clinic had higher probability for working full-time during the first years of follow-up, than those being on full-time sick leave.

Our findings are in line with other studies showing that RTW can be a long and resource consuming process [19,25]. It also emphasises the need for analyses of multiple, longitudinal and repeated events for a better understanding of the RTW process [3,5,26]. RTW is not an absorbing state and cannot be measured at a single point of time [25,26]. Multi-state models, as used in our study, can be a useful method to analyze longitudinal RTW data. However there is no clear agreement about how long of a follow-up period is needed to get the best measurement of the effect on work and benefits, after sick leave and work-related interventions [27,28]. Our data indicate that several years are needed to get an adequate picture of the RTW outcome. Differences in design, populations and follow-up period in RTW studies make it difficult to compare results between studies [1,27,28].

Our data also demonstrates the importance of including information on partial sick leave benefits and part-time work, when evaluating RTW after rehabilitation programmes [29]. This is in accordance with recommendations in research on sick leave [1,5,19]. For individuals on long-term sick leave, with limited ability to function, working part-time may be a sufficient goal after rehabilitation, especially when the alternative would be full-time disability pension. Since work ability is not a fixed either/or category, but changes over time, and with varying circumstances, working part-time may also play a role in transitions to full-time employment [30]. It may therefore be important to have a flexible working life, giving opportunities for part-time work for workers with temporary low work ability [30].

We found an increased probability for working after receiving either medical or vocational rehabilitation allowances, at the time of departure from a rehabilitation clinic. This finding is in accordance with the idea behind the legal regulations for sick leave benefits in Norway. Medical rehabilitation allowances should only be granted to individuals with treatable medical conditions, whereas vocational rehabilitation allowance is restricted to individuals with an expected likelihood for RTW after e.g. work training or professional re-education. After the first year on sick leave benefits, medical and vocational rehabilitation allowances constitute a secure source of income for long-term sick workers in Norway. These allowances are equivalent to the sickness benefits system in Sweden before 2010, where sick leave benefits could be expanded beyond the first year in certain medical cases [5].

However receiving such benefits has been claimed to be a step toward disability pension, indicating that RTW is difficult to achieve after more than one year out of work. In Sweden the sick leave track record was found to be the most important predictor of the probability of being granted a disability pension [31,32]. The probability of being granted disability pension following sick leave often begins with short term sick leave periods followed by episodes increasing in length, and with shorter and shorter intervals between them [31]. Few studies have explored outcomes after such allowances in Norway, but Landstad and co-authors [33] found that only 27% returned to work after receiving medical rehabilitation allowance. RTW was, among other factors, associated with prior work life affiliation, contact and satisfaction with the work place during rehabilitation, and experiencing a high degree of influence over their own rehabilitation process [33]. Even though this was not an intervention study, the findings may have relevance in the interpretation of the optimistic RTW outcomes in our study. The rehabilitation program emphasized client participation and contact with the workplace, and the final part of the intervention was to agree on a RTW plan. Workplace engagement, in terms of coordination and cooperation between client, employer and different stakeholders involved in the RTW process is stated to be crucial for success in re-integrating individuals on sick leave in working life [3,20,34-36]. Offering adequate rehabilitation efforts may alter a negative trend out of working life when it is given to the right type of patients.

Furthermore we found an increase in medical and vocational rehabilitation allowance and a simultaneously decrease in sick leave benefit during the first months after the rehabilitation program. This may be interpreted as an effect of the social security legislation on time limits for sick leave benefit to maximum one year continuously. As the participants on average already had been out of work for 9 months before the rehabilitation stay, some were about to cross the time limit at departure from the clinic. These legislations, and the decrease in social security disbursement to 66% of the salary after one year, may also be an important incentive to RTW.

The current study adds to previous literature by a detailed analysis exploring the RTW process and pathways back to and out of work after work-related rehabilitation. To our knowledge, a multistate model on sick leave data has not earlier included this amount of possible outcomes. Lie et al. (2008) restricted their analysis to three different states, in their follow-up of low back pain patients in Norway [1]. Analysis on register data in the Nordic countries has been restricted to sick leave benefits and disability pension [1] and to unemployment benefits [32].

The use of social security benefits and RTW is a frequent issue in public health research, irrespective of nationality [31,37]. Comparison between studies may be difficult due to the large variation between countries in the regulation of sick leave compensations and the granting of disability pension [31]. Within the Nordic countries the social security system, representing The Nordic welfare model, is more similar, and makes comparison between the Nordic countries more suitable [5,27,37,38]. These countries are facing the same challenges towards undesirable high rates of sick leave and disability pension [5]. The countries are also somewhat similar in the access to official registers, making it possible to follow individuals on sick leave through the social security system [31]. Our findings may therefore be generalized to the Nordic countries.

Despite the strength of using register data in the long term follow-up of after rehabilitation, there are some limitations in this study. Register data from The National Labour and Welfare in Norway contains spare information on whether a person is actually working or not. Hence we assumed that individuals were working in the time gaps between dates of benefits in the register. This is believed to be a correct interpretation of such registers since people that support themselves without a job usually have no legal rights to receive sick leave benefits [27]. Based on our analysis, we believe that the definition of work, being the time gaps in the register files, is valid. It was unusually many transitions from work directly to disability pension. This may be explained by the delays in the case procedures within The National Labour and Welfare system. Disability pension claimants may be without registered benefits in a short period while waiting for a decision. Data on unemployment benefits would have strengthened the study further. However the number of people on unemployment benefits in Norway is low.

## Conclusions

The process to RTW or to receive disability pension was complex, and may take several years, with multiple transitions between work and different benefits. The multistate RTW model, analysing official register data in the follow-up of individuals on long-term sick leave, offers useful information about the long-lasting and complex recovery and disability process after a rehabilitation program. Further research is needed to explore individual characteristics and environmental factors associated with different transitions and pathways out of or back to work.

## Competing interests

The authors declare that they have no conflicts of interests.

## Authors’ contributions

All authors participated in the design of the study, helped draft the manuscript and to interpret the results. IØ was involved in the planning of the study, in the data collection, in the statistical analysis, and drafted the manuscript. SAL was involved in the data collection, carried out all the statistical analysis, and gave structural advice in all parts of the study. CMI gave important advice and contributed continuously in the writing process. HRE was involved in the planning of the study, gave critical advice and contributed in all parts of the study. All authors read and approved the final manuscript.

## Pre-publication history

The pre-publication history for this paper can be accessed here:

http://www.biomedcentral.com/1471-2458/12/748/prepub

## Author details

^1^The National Centre for Occupational Rehabilitation, Haddlandvegen 20, 3864 Rauland, Norway. ^2^Uni Health, Uni Research, Bergen, Norway. ^3^Health UMB, IHA, University of Life Sciences, Ås, Norway. ^4^Department of Health Promotion and Development, University of Bergen, Bergen, Norway.
